# (*E*)-*N*′-(5-Bromo-2-hydroxy­benzyl­idene)-3,4,5-trimethoxy­benzohydrazide

**DOI:** 10.1107/S1600536808038464

**Published:** 2008-11-22

**Authors:** Zhen-dong Zhao, Yu-min Wang, Yu-xiang Chen, Liang-wu Bi

**Affiliations:** a Institute of Chemical Industry of Forest Products, Chinese Academy of Forestry, Nanjing 210042, People’s Republic of China

## Abstract

The mol­ecule of the title compound, C_17_H_17_BrN_2_O_5_, assumes an *E* configuration, with the 5-bromo-2-hydroxy­phenyl and benzohydrazide units located on opposite sites of the C=N double bond. The dihedral angle between the planes of the two benzene rings is 32.48 (15)°. The crystal structure is stabilized by intra­molecular O—H⋯N and inter­molecular N—H⋯O hydrogen bonds.

## Related literature

For related literature, see: Yang *et al.* (1996 ▶); Nawar & Hosny (2000 ▶); Pelagatti *et al.* (1999 ▶); Ainscough *et al.* (1998 ▶). For related structures, see: Diao & Yu (2006 ▶); Jing *et al.* (2005 ▶); Wang *et al.* (2008 ▶).
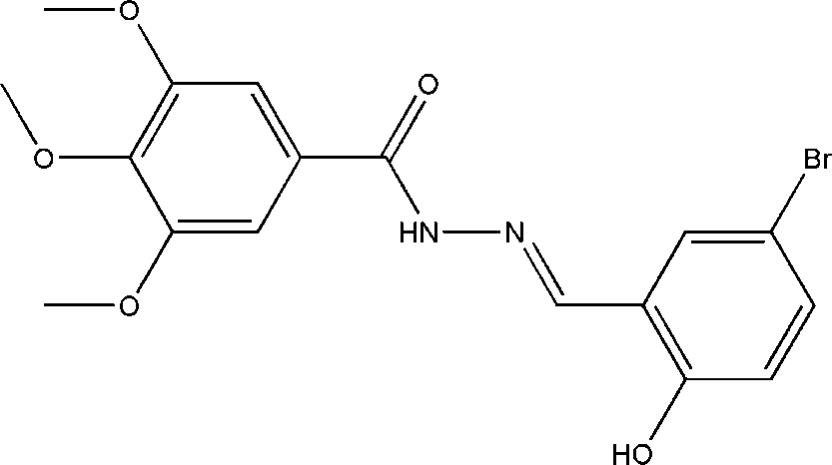


## Experimental

### 

#### Crystal data

C_17_H_17_BrN_2_O_5_
*M* *_r_* = 409.24
Monoclinic, 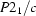

*a* = 11.4157 (19) Å
*b* = 16.279 (3) Å
*c* = 9.3738 (16) Å
β = 100.210 (3)°
*V* = 1714.4 (5) Å^3^
*Z* = 4
Mo *K*α radiation
μ = 2.43 mm^−1^
*T* = 273 (2) K [Unusual. Please check]
0.15 × 0.10 × 0.06 mm

#### Data collection

Bruker APEX CCD area-detector diffractometer
Absorption correction: multi-scan (*SADABS*; Sheldrick, 1996 ▶) *T*_min_ = 0.712, *T*_max_ = 0.868
8954 measured reflections
3037 independent reflections
2065 reflections with *I* > 2σ(*I*)
*R*_int_ = 0.033

#### Refinement

*R*[*F* ^2^ > 2σ(*F* ^2^)] = 0.037
*wR*(*F* ^2^) = 0.097
*S* = 1.01
3037 reflections
228 parameters
H-atom parameters constrained
Δρ_max_ = 0.43 e Å^−3^
Δρ_min_ = −0.54 e Å^−3^

### 

Data collection: *SMART* (Bruker, 1997 ▶); cell refinement: *SAINT* (Bruker, 1997 ▶); data reduction: *SAINT*; program(s) used to solve structure: *SHELXS97* (Sheldrick, 2008 ▶); program(s) used to refine structure: *SHELXL97* (Sheldrick, 2008 ▶); molecular graphics: *SHELXTL* (Sheldrick, 2008 ▶); software used to prepare material for publication: *SHELXTL*.

## Supplementary Material

Crystal structure: contains datablocks I, global. DOI: 10.1107/S1600536808038464/pv2120sup1.cif

Structure factors: contains datablocks I. DOI: 10.1107/S1600536808038464/pv2120Isup2.hkl

Additional supplementary materials:  crystallographic information; 3D view; checkCIF report

## Figures and Tables

**Table 1:** Hydrogen-bond geometry (Å, °)

*D*—H⋯*A*	*D*—H	H⋯*A*	*D*⋯*A*	*D*—H⋯*A*
O1—H1⋯N2	0.82	1.92	2.642 (3)	145
N1—H1*A*⋯O2^i^	0.86	2.14	2.888 (3)	146

Symmetry code: (i) 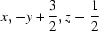.

**Fig. 1.**
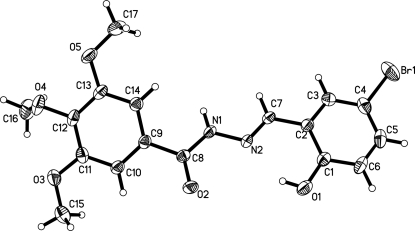
A view of the molecular structure of (I), showing displacement ellipsoids at the 50% probability level.

**Fig. 2.**
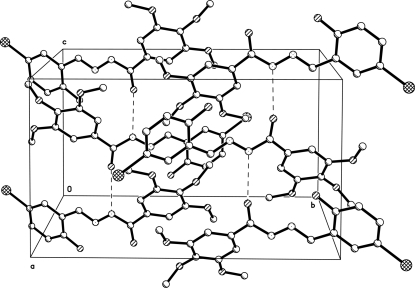
The packing of (I), showing the intermolecular hydrogen-bonded extended network.

## supplementary crystallographic information

### Comment

Salicyladelhyde hydrazones have received considerable attention because of their possible applications in catalysis and pharmacology (Yang *et al.*, 1996; Nawar & Hosny, 2000; Pelagatti *et al.*, 1999; Ainscough *et al.*, 1998). As part of our ongoing investigation on acylhydrazone compounds (Wang *et al.*, 2008) and their metal complexes, we have obtained the title compound, (I), from a condensation of 3,4,5-trimethoxybenzohydrazide and 5-bromo-2-hydroxybenzaldehyde, and report its crystal structure in this paper.

The molecular structure of (I) (Fig. 1) contains 3,4,5-trimethoxybenzohydrazide and 5-bromo-2-hydroxybenzaldehyde moities that are located on opposite sides of the C═N double bond exhibiting an E configuration about C═N. The dihedral angle between the mean-planes of the two benzene rings is 32.48 (15)°. The crystal structure involves an intramolecular hydrogen bond O1–H1···N2, and is further stabilized by an intermolecular hydrogen bonds N1–H1A···O2, resulting in chains of molecules extended along the *c*-axis; details are given in Table 1 and Fig. 2. The bond distances and bond angles in (I) are in agreement with the corresponding dimensions reported for some structures closely related to (I) (Diao & Yu, 2006; Jing *et al.*, 2005; Wang *et al.*, 2008). (

### Experimental

An ethanol solution (50 ml) of 3,4,5-trimethoxybenzohydrazide (0.01 mol) and 5-bromo-2-hydroxybenzaldehyde (0.01 mol) was refluxed and stirred for 2h ; the mixture was cooled and the resulting solid product, (I), was collected by filtration. Crystals suitable for single-crystal X-ray diffraction were grown by slow evaporation of a solution in THF.

### Refinement

All H atoms  were placed geometrically at the distances: C—H(methyl) = 0.96, C—H(aryl) = 0.93, N—H = 0.86 and O—H = 0.82 Å and included in the refinement in riding motion approximation with U_iso_(H) = 1.2 or 1.5U_eq_ of the carrier atom.

### Crystal data

**Table:**

C_17_H_17_BrN_2_O_5_	*F*_000_ = 832
*M**_r_* = 409.24	*D*_x_ = 1.586 Mg m^−^^3^
Monoclinic, *P*2_1_/*c*	Mo *K*α radiation λ = 0.71073 Å
Hall symbol: -P 2ybc	Cell parameters from 2452 reflections
*a* = 11.4157 (19) Å	θ = 2.5–22.8º
*b* = 16.279 (3) Å	µ = 2.43 mm^−^^1^
*c* = 9.3738 (16) Å	*T* = 273 (2) K
β = 100.210 (3)º	Block, colourless
*V* = 1714.4 (5) Å^3^	0.15 × 0.10 × 0.06 mm
*Z* = 4	

### Data collection

**Table:**

Bruker APEX CCD area-detector diffractometer	3037 independent reflections
Radiation source: fine-focus sealed tube	2065 reflections with *I* > 2σ(*I*)
Monochromator: graphite	*R*_int_ = 0.033
*T* = 273(2) K	θ_max_ = 25.1º
φ and ω scans	θ_min_ = 1.8º
Absorption correction: multi-scan(SADABS; Sheldrick, 1996)	*h* = −13→12
*T*_min_ = 0.712, *T*_max_ = 0.868	*k* = −19→19
8954 measured reflections	*l* = −11→8

### Refinement

**Table:**

Refinement on *F*^2^	Hydrogen site location: inferred from neighbouring sites
Least-squares matrix: full	H-atom parameters constrained
*R*[*F*^2^ > 2σ(*F*^2^)] = 0.037	*w* = 1/[σ^2^(*F*_o_^2^) + (0.0439*P*)^2^ + 0.6769*P*] where *P* = (*F*_o_^2^ + 2*F*_c_^2^)/3
*wR*(*F*^2^) = 0.097	(Δ/σ)_max_ = 0.002
*S* = 1.01	Δρ_max_ = 0.43 e Å^−^^3^
3037 reflections	Δρ_min_ = −0.54 e Å^−^^3^
228 parameters	Extinction correction: SHELXL97 (Sheldrick, 2008), Fc^*^=kFc[1+0.001xFc^2^λ^3^/sin(2θ)]^-1/4^
Primary atom site location: structure-invariant direct methods	Extinction coefficient: 0.0171 (11)
Secondary atom site location: difference Fourier map	

### Special details

**Table:**

Geometry. All e.s.d.'s (except the e.s.d. in the dihedral angle between two l.s. planes)are estimated using the full covariance matrix. The cell e.s.d.'s are takeninto account individually in the estimation of e.s.d.'s in distances, anglesand torsion angles; correlations between e.s.d.'s in cell parameters are onlyused when they are defined by crystal symmetry. An approximate (isotropic)treatment of cell e.s.d.'s is used for estimating e.s.d.'s involving l.s. planes.
Refinement. Refinement of *F*^2^ against ALL reflections. The weighted *R*-factor *wR* andgoodness of fit *S* are based on *F*^2^, conventional *R*-factors *R* are basedon *F*, with *F* set to zero for negative *F*^2^. The threshold expression of*F*^2^ > σ(*F*^2^) is used only for calculating *R*-factors(gt) *etc*. and isnot relevant to the choice of reflections for refinement. *R*-factors basedon *F*^2^ are statistically about twice as large as those based on *F*, and *R*-factors based on ALL data will be even larger.

### Fractional atomic coordinates and isotropic or equivalent isotropic displacement parameters (Å^2^)

**Table:**

	*x*	*y*	*z*	*U*_iso_*/*U*_eq_	
Br1	0.94048 (4)	1.21831 (2)	0.94475 (6)	0.0865 (2)	
O1	0.9086 (2)	0.92631 (14)	1.3259 (2)	0.0609 (6)	
H1	0.8677	0.8897	1.2824	0.091*	
O2	0.74380 (19)	0.70776 (12)	1.2188 (2)	0.0493 (5)	
O3	0.6562 (2)	0.42963 (12)	0.9513 (2)	0.0605 (6)	
O4	0.4797 (2)	0.47058 (14)	0.7287 (2)	0.0661 (7)	
O5	0.42036 (19)	0.62400 (14)	0.6698 (2)	0.0595 (6)	
N1	0.7240 (2)	0.78966 (13)	1.0217 (2)	0.0413 (6)	
H1A	0.6980	0.7944	0.9302	0.050*	
N2	0.7787 (2)	0.85483 (14)	1.0984 (2)	0.0392 (6)	
C1	0.9120 (3)	0.99080 (18)	1.2363 (3)	0.0446 (7)	
C2	0.8549 (2)	0.98993 (16)	1.0913 (3)	0.0400 (7)	
C3	0.8637 (3)	1.05886 (18)	1.0069 (3)	0.0485 (8)	
H3	0.8252	1.0596	0.9107	0.058*	
C4	0.9282 (3)	1.12578 (18)	1.0634 (4)	0.0542 (8)	
C5	0.9863 (3)	1.1252 (2)	1.2051 (4)	0.0592 (9)	
H5	1.0316	1.1702	1.2427	0.071*	
C6	0.9772 (3)	1.0587 (2)	1.2899 (4)	0.0573 (9)	
H6	1.0158	1.0591	1.3861	0.069*	
C7	0.7914 (2)	0.91924 (17)	1.0253 (3)	0.0417 (7)	
H7	0.7588	0.9206	0.9271	0.050*	
C8	0.7105 (2)	0.71797 (17)	1.0892 (3)	0.0369 (6)	
C9	0.6508 (2)	0.65335 (17)	0.9912 (3)	0.0381 (7)	
C10	0.6826 (3)	0.57283 (17)	1.0217 (3)	0.0416 (7)	
H10	0.7397	0.5600	1.1023	0.050*	
C11	0.6292 (3)	0.51139 (17)	0.9317 (3)	0.0450 (7)	
C12	0.5412 (3)	0.52992 (18)	0.8141 (3)	0.0460 (7)	
C13	0.5089 (3)	0.61112 (19)	0.7852 (3)	0.0445 (7)	
C14	0.5643 (2)	0.67246 (18)	0.8733 (3)	0.0413 (7)	
H14	0.5434	0.7270	0.8534	0.050*	
C15	0.7519 (3)	0.4079 (2)	1.0611 (4)	0.0713 (10)	
H15A	0.8233	0.4340	1.0431	0.107*	
H15B	0.7622	0.3493	1.0618	0.107*	
H15C	0.7355	0.4255	1.1533	0.107*	
C16	0.5452 (4)	0.4238 (2)	0.6446 (4)	0.0853 (12)	
H16A	0.5791	0.4595	0.5812	0.128*	
H16B	0.4935	0.3848	0.5880	0.128*	
H16C	0.6077	0.3952	0.7071	0.128*	
C17	0.3907 (3)	0.7059 (2)	0.6315 (4)	0.0697 (10)	
H17A	0.3538	0.7308	0.7052	0.105*	
H17B	0.3364	0.7070	0.5407	0.105*	
H17C	0.4616	0.7357	0.6225	0.105*	

### Atomic displacement parameters (Å^2^)

**Table:**

	*U*^11^	*U*^22^	*U*^33^	*U*^12^	*U*^13^	*U*^23^
Br1	0.0686 (3)	0.0506 (3)	0.1438 (5)	0.00267 (18)	0.0284 (3)	0.0265 (2)
O1	0.0746 (17)	0.0646 (15)	0.0377 (12)	−0.0140 (12)	−0.0059 (11)	−0.0017 (11)
O2	0.0660 (14)	0.0498 (12)	0.0287 (11)	0.0048 (10)	−0.0008 (10)	−0.0018 (9)
O3	0.0799 (16)	0.0411 (12)	0.0584 (14)	−0.0084 (11)	0.0066 (13)	−0.0024 (11)
O4	0.0701 (16)	0.0687 (15)	0.0606 (15)	−0.0312 (12)	0.0147 (13)	−0.0247 (12)
O5	0.0551 (14)	0.0737 (16)	0.0426 (13)	−0.0110 (12)	−0.0106 (11)	−0.0072 (11)
N1	0.0504 (14)	0.0429 (14)	0.0266 (12)	−0.0088 (11)	−0.0039 (11)	−0.0032 (11)
N2	0.0450 (14)	0.0359 (13)	0.0336 (13)	−0.0029 (11)	−0.0019 (11)	−0.0071 (11)
C1	0.0470 (17)	0.0485 (18)	0.0377 (17)	−0.0016 (14)	0.0053 (15)	−0.0063 (14)
C2	0.0383 (16)	0.0407 (16)	0.0394 (17)	0.0028 (13)	0.0032 (14)	−0.0047 (13)
C3	0.0459 (18)	0.0463 (18)	0.0512 (19)	0.0034 (14)	0.0031 (15)	0.0013 (15)
C4	0.0466 (19)	0.0399 (17)	0.079 (3)	0.0017 (14)	0.0184 (19)	0.0001 (17)
C5	0.051 (2)	0.0457 (19)	0.082 (3)	−0.0077 (15)	0.0138 (19)	−0.0220 (19)
C6	0.052 (2)	0.063 (2)	0.054 (2)	−0.0096 (16)	0.0002 (16)	−0.0239 (18)
C7	0.0454 (17)	0.0456 (17)	0.0304 (15)	−0.0010 (14)	−0.0032 (13)	−0.0056 (14)
C8	0.0372 (15)	0.0422 (16)	0.0308 (16)	0.0025 (13)	0.0051 (13)	−0.0042 (13)
C9	0.0416 (16)	0.0406 (16)	0.0325 (15)	−0.0050 (13)	0.0077 (13)	−0.0048 (13)
C10	0.0436 (17)	0.0463 (17)	0.0339 (16)	−0.0040 (14)	0.0043 (13)	−0.0037 (13)
C11	0.0531 (19)	0.0387 (16)	0.0471 (18)	−0.0075 (14)	0.0194 (16)	−0.0045 (14)
C12	0.0483 (18)	0.0507 (18)	0.0400 (18)	−0.0182 (15)	0.0107 (15)	−0.0130 (15)
C13	0.0396 (17)	0.060 (2)	0.0333 (17)	−0.0103 (14)	0.0033 (14)	−0.0069 (14)
C14	0.0429 (17)	0.0436 (17)	0.0371 (16)	−0.0028 (13)	0.0064 (14)	−0.0015 (13)
C15	0.084 (3)	0.0450 (19)	0.083 (3)	0.0038 (18)	0.010 (2)	0.0028 (18)
C16	0.113 (3)	0.066 (2)	0.075 (3)	−0.008 (2)	0.011 (3)	−0.035 (2)
C17	0.056 (2)	0.084 (3)	0.061 (2)	−0.0003 (19)	−0.0123 (18)	0.004 (2)

### Geometric parameters (Å, °)

**Table:**

Br1—C4	1.892 (3)	C5—H5	0.9300
O1—C1	1.350 (4)	C6—H6	0.9300
O1—H1	0.8200	C7—H7	0.9300
O2—C8	1.219 (3)	C8—C9	1.481 (4)
O3—C11	1.371 (3)	C9—C10	1.377 (4)
O3—C15	1.407 (4)	C9—C14	1.381 (4)
O4—C12	1.366 (3)	C10—C11	1.379 (4)
O4—C16	1.403 (4)	C10—H10	0.9300
O5—C13	1.359 (3)	C11—C12	1.386 (4)
O5—C17	1.406 (4)	C12—C13	1.386 (4)
N1—C8	1.349 (3)	C13—C14	1.376 (4)
N1—N2	1.369 (3)	C14—H14	0.9300
N1—H1A	0.8600	C15—H15A	0.9600
N2—C7	1.274 (3)	C15—H15B	0.9600
C1—C6	1.378 (4)	C15—H15C	0.9600
C1—C2	1.399 (4)	C16—H16A	0.9600
C2—C3	1.387 (4)	C16—H16B	0.9600
C2—C7	1.440 (4)	C16—H16C	0.9600
C3—C4	1.368 (4)	C17—H17A	0.9600
C3—H3	0.9300	C17—H17B	0.9600
C4—C5	1.375 (5)	C17—H17C	0.9600
C5—C6	1.357 (5)		
			
C1—O1—H1	109.5	C14—C9—C8	121.4 (3)
C11—O3—C15	118.0 (2)	C9—C10—C11	119.4 (3)
C12—O4—C16	116.3 (3)	C9—C10—H10	120.3
C13—O5—C17	117.4 (2)	C11—C10—H10	120.3
C8—N1—N2	120.2 (2)	O3—C11—C10	123.9 (3)
C8—N1—H1A	119.9	O3—C11—C12	115.6 (3)
N2—N1—H1A	119.9	C10—C11—C12	120.5 (3)
C7—N2—N1	116.1 (2)	O4—C12—C13	117.9 (3)
O1—C1—C6	118.1 (3)	O4—C12—C11	122.4 (3)
O1—C1—C2	122.5 (3)	C13—C12—C11	119.5 (3)
C6—C1—C2	119.4 (3)	O5—C13—C14	124.3 (3)
C3—C2—C1	118.5 (3)	O5—C13—C12	115.9 (3)
C3—C2—C7	118.9 (3)	C14—C13—C12	119.8 (3)
C1—C2—C7	122.5 (3)	C13—C14—C9	120.3 (3)
C4—C3—C2	120.8 (3)	C13—C14—H14	119.9
C4—C3—H3	119.6	C9—C14—H14	119.9
C2—C3—H3	119.6	O3—C15—H15A	109.5
C3—C4—C5	120.2 (3)	O3—C15—H15B	109.5
C3—C4—Br1	119.8 (3)	H15A—C15—H15B	109.5
C5—C4—Br1	120.0 (3)	O3—C15—H15C	109.5
C6—C5—C4	119.9 (3)	H15A—C15—H15C	109.5
C6—C5—H5	120.1	H15B—C15—H15C	109.5
C4—C5—H5	120.1	O4—C16—H16A	109.5
C5—C6—C1	121.2 (3)	O4—C16—H16B	109.5
C5—C6—H6	119.4	H16A—C16—H16B	109.5
C1—C6—H6	119.4	O4—C16—H16C	109.5
N2—C7—C2	121.6 (3)	H16A—C16—H16C	109.5
N2—C7—H7	119.2	H16B—C16—H16C	109.5
C2—C7—H7	119.2	O5—C17—H17A	109.5
O2—C8—N1	123.0 (2)	O5—C17—H17B	109.5
O2—C8—C9	123.3 (3)	H17A—C17—H17B	109.5
N1—C8—C9	113.7 (2)	O5—C17—H17C	109.5
C10—C9—C14	120.4 (3)	H17A—C17—H17C	109.5
C10—C9—C8	118.2 (3)	H17B—C17—H17C	109.5

### Hydrogen-bond geometry (Å, °)

**Table:**

*D*—H···*A*	*D*—H	H···*A*	*D*···*A*	*D*—H···*A*
O1—H1···N2	0.82	1.92	2.642 (3)	145
N1—H1A···O2^i^	0.86	2.14	2.888 (3)	146

Symmetry codes: (i) *x*, −*y*+3/2, *z*−1/2.

## References

[bb1] Ainscough, E. W., Brodie, A. M., Dobbs, A. J., Ranford, J. D. & Waters, J. M. (1998). *Inorg Chim. Acta*, **267**, 27–38.

[bb2] Bruker (1997). *SMART* and *SAINT* Bruker AXS Inc., Madison, Wisconsin, USA.

[bb3] Diao, C.-H. & Yu, M. (2006). *Acta Cryst.* E**62**, o5278–o5279.

[bb4] Jing, Z.-L., Fan, Z., Yu, M., Chen, X. & Deng, Q.-L. (2005). *Acta Cryst.* E**61**, o3495–o3496.

[bb5] Nawar, N. & Hosny, N. M. (2000). *Transition Met. Chem.* **25**, 1–8.

[bb6] Pelagatti, P., Bacchi, A., Carcelli, M., Costa, M., Fochi, A., Ghidini, P., Leporati, E., Masi, M., Pelizzi, C. & Pelizzi, G. (1999). *J. Organomet. Chem.* **583**, 94–105.

[bb7] Sheldrick, G. M. (1996). *SADABS* University of Göttingen, Germany.

[bb8] Sheldrick, G. M. (2008). *Acta Cryst.* A**64**, 112–122. 10.1107/S0108767307043930 18156677

[bb9] Wang, Y.-M., Zhao, Z.-D., Chen, Y.-X. & Bi, L.-W. (2008). *Acta Cryst.* E**64**, o1009. 10.1107/S1600536808006077 PMC2961635 21202535

[bb10] Yang, Z. Y., Yang, R. D. & Yu, K. B. (1996). *Polyhedron*, **15**, 3749–3753.

